# Regional Gas Exchange Measured by ^129^Xe Magnetic Resonance Imaging Before and After Combination Bronchodilators Treatment in Chronic Obstructive Pulmonary Disease

**DOI:** 10.1002/jmri.27662

**Published:** 2021-05-07

**Authors:** David G. Mummy, Erika M. Coleman, Ziyi Wang, Elianna A. Bier, Junlan Lu, Bastiaan Driehuys, Yuh‐Chin Huang

**Affiliations:** ^1^ Center for In Vivo Microscopy Duke University Durham North Carolina USA; ^2^ Department of Radiology Duke University Medical Center Durham North Carolina USA; ^3^ Department of Medicine Duke University Medical Center Durham North Carolina USA; ^4^ Department of Biomedical Engineering Duke University Durham North Carolina USA; ^5^ Department of Medical Physics Duke University Durham North Carolina USA

**Keywords:** xenon‐129, magnetic resonance imaging, lung, bronchodilator agents, chronic obstructive pulmonary disease

## Abstract

**Background:**

Hyperpolarized ^129^Xe magnetic resonance imaging (MRI) provides a non‐invasive assessment of regional pulmonary gas exchange function. This technique has demonstrated that chronic obstructive pulmonary disease (COPD) patients exhibit ventilation defects, reduced interstitial barrier tissue uptake, and poor transfer to capillary red blood cells (RBCs). However, the behavior of these measurements following therapeutic intervention is unknown.

**Purpose:**

To characterize changes in ^129^Xe gas transfer function following administration of an inhaled long‐acting beta‐agonist/long‐acting muscarinic receptor antagonist (LABA/LAMA) bronchodilator.

**Study Type:**

Prospective.

**Population:**

Seventeen COPD subjects (GOLD II/III classification per Global Initiative for Chronic Obstructive Lung Disease criteria) were imaged before and after 2 weeks of LABA/LAMA therapy.

**Field Strength/Sequences:**

Dedicated ventilation imaging used a multi‐slice 2D gradient echo sequence. Three‐dimensional images of ventilation, barrier uptake, and RBC transfer used an interleaved, radial, 1‐point Dixon sequence. Imaging was acquired at 3 T.

**Assessment:**

^129^Xe measurements were quantified before and after LABA/LAMA treatment by ventilation defect + low percent (ven_def + low_) and by barrier uptake and RBC transfer relative to a healthy reference population (bar_%ref_ and RBC_%ref_). Pulmonary function tests, including diffusing capacity of the lung for carbon monoxide (DL_CO_), were also performed before and after treatment.

**Statistical Tests:**

Paired *t*‐test, Pearson correlation coefficient (*r*).

**Results:**

Baseline ven_def + low_ was 57.8 ± 8.4%, bar_%ref_ was 73.2 ± 19.6%, and RBC_%ref_ was 36.5 ± 13.6%. Following treatment, ven_def + low_ decreased to 52.5 ± 10.6% (*P* < 0.05), and improved in 14/17 (82.4%) of subjects. However, RBC_%ref_ decreased in 10/17 (58.8%) of subjects. Baseline measurements of bar_%ref_ and DL_CO_ were correlated with the degree of post‐treatment change in ven_def + low_ (*r* = −0.49, *P* < 0.05 and *r* = −0.52, *P* < 0.05, respectively).

**Conclusion:**

LABA/LAMA therapy tended to preferentially improve ventilation in subjects whose ^129^Xe barrier uptake and DL_CO_ were relatively preserved. However, newly ventilated regions often revealed RBC transfer defects, an aspect of lung function opaque to spirometry. These microvasculature abnormalities must be accounted for when assessing the effects of LABA/LAMA therapy.

**Level of Evidence:**

1

**Technical Efficacy Stage:**

4

Hyperpolarized (HP) ^129^Xe gas magnetic resonance imaging (MRI) is a promising tool for 3D visualization and quantification of regional gas exchange function in the lung. It offers faster imaging and superior spatial resolution compared to the radionuclide ventilation‐perfusion scan. Such functional pulmonary MRI techniques can complement the detailed structural information provided by computed tomography (CT), and because they do not expose patients to ionizing radiation, can be performed as a means of longitudinal monitoring.^1^

HP ^129^Xe is sensitive to ventilation defects in both chronic obstructive pulmonary disease (COPD)^2^, ^3^ and asthma.^4^ In asthma, it has been used to visualize the regional reversal of defects after bronchodilator administration.^5^ Beyond ventilation imaging, ^129^Xe MRI also provides the unique ability to image the diffusive transfer of gas into the alveolar‐capillary barrier tissue and red blood cell (RBC) compartments^6^ and recent work has provided a model to relate this technique to the observed DL_CO_.^7^ ^129^Xe MRI has been used to demonstrate that patients with idiopathic pulmonary fibrosis (IPF) exhibit increased ^129^Xe uptake into the barrier tissues consistent with thickened lung interstitium, and these ^129^Xe‐MRI‐derived metrics correlated significantly with pulmonary function tests (PFTs) but not with CT fibrosis scores.^8^ Notably, the combination of both increased barrier uptake and poor RBC transfer has been associated with poor outcomes in patients with IPF.^9^ ^129^Xe gas exchange MRI in COPD patients has demonstrated a combination of ventilation defects, low barrier uptake, and poor transfer of ^129^Xe to RBCs compared to other lung diseases.^10^ However, ^129^Xe MRI measures of gas transfer in COPD have not been evaluated in the context of a therapeutic intervention. We thus have little understanding of which measures are affected by therapy or whether any markers of ^129^Xe gas exchange are predictive of positive therapy response.

Among the major treatment options for COPD are the dual bronchodilator preparations that have been shown to increase forced expiratory volume in 1 second (FEV_1_) and decrease the frequency of acute exacerbation.^11^, ^12^ However, it remains unclear how improvement in FEV_1_, a global measurement of lung mechanics, may affect regional gas exchange function.

Thus, the aim of this study was to use HP ^129^Xe MRI to assess gas transfer function in patients with COPD before and after treatment with an inhaled long‐acting beta‐agonist/long‐acting muscarinic receptor antagonist (LABA/LAMA) bronchodilator preparation (glycopyrrolate/formoterol fumarate). We tested whether baseline measurements, including clinical variables, spirometry, and ^129^Xe gas exchange, were predictive of ventilation improvement as measured using ^129^Xe MRI following the dual LABA/LAMA bronchodilator preparation.

## Materials and Methods

### Patient Population

We recruited 17 subjects age ≥40 of any sex with a pulmonologist diagnosis of COPD. Diagnosis was based on the clinical and spirometric criteria stated in the Global Initiative for Chronic Obstructive Lung Disease (GOLD) 2017 guidelines^13^: dyspnea, chronic cough or sputum production, a history of recurrent lower respiratory tract infections, and/or a history of exposure to risk factors for the disease, together with a post‐bronchodilator FEV_1_/FVC < 0.70. Subjects were classified as either GOLD II (FEV_1_ 50–79%) or GOLD III (FEV_1_ 30–49%). All subjects had a smoking history of at least 10 pack‐years.

Subjects were required to be at least 6 weeks removed from an upper respiratory tract infection or acute exacerbation. The following exclusion criteria were applied: 1) daily use of >10 mg of systemic steroids; 2) chronic oxygen therapy; 3) a history of lung surgery including resection, decortication, or pneumothorax; 4) diagnosis of asthma‐COPD overlap syndrome; 5) a history of exposure to occupational hazards known to cause lung disease; 6) a history of myocardial infarction, unstable angina, cardiac arrhythmias, cardiomyopathy, uncontrolled hypertension, uncontrolled diabetes mellitus, diabetic ketoacidosis, thyrotoxicosis, seizures, hypokalemia, and/or narrow‐angle glaucoma; 7) interstitial or chronic infectious lung disease confirmed by imaging studies; and/or 8) pregnancy.

### Protocol

All participants signed a written, informed consent prior to enrollment and the HIPAA‐compliant study protocol was approved by the Institutional Review Board of Duke University. All subjects who were on regular inhalation therapies for COPD underwent a washout period of 7–10 days, during which they stopped all inhaled corticosteroids, LABA and LAMA. Use of inhaled albuterol was allowed as needed. At the end of the washout period, subjects received a pre‐treatment ^129^Xe MRI scan and PFTs together with an assessment of secondary outcomes (described below). They were then given a two‐week sample of glycopyrrolate/formoterol aerosphere (Bevespi®, AstraZeneca, Cambridge, United Kingdom) at a dose of 2 puffs twice a day, and were trained on correct administration technique. After 2 weeks of therapy, subjects returned for a post‐treatment ^129^Xe MRI scan, PFTs, and assessment of secondary outcomes. A follow‐up phone call was conducted after an additional 2‐week period to assess any potential adverse events.

### ^129^Xe Polarization and Dose Administration

^129^Xe was hyperpolarized via continuous flow spin‐exchange optical pumping and cryogenic accumulation using commercially available systems (Model 9820 and 9810; Polarean plc., Durham, NC) and dispensed into a Tedlar™ dose delivery bag as previously described.^14^ HP ^129^Xe imaging and spectroscopy were acquired on a 3‐T scanner (Magnetom Trio; Siemens, Erlangen, Germany) during three separate breath‐holds, one each for calibration, dedicated ventilation, and gas exchange scans. Subjects received a small dose (target ^129^Xe dose equivalent [DE] ≥ 65, actual 64.9 ± 16.8 mL) for calibration followed by two larger doses for the gas exchange (target DE ≥150 mL, actual 180.9 ± 33.1 mL) and dedicated ventilation scans (DE ≥80 mL, actual 96.5 ± 23.2 mL) with the scans performed in that order. The dose equivalent represents the magnetization that would be provided by the indicated volume of 100% enriched, 100% polarized ^129^Xe.^14^ These dose equivalents were achieved with xenon volumes ranging from 250 mL to 715 mL expanded with the same 89% helium blend used in ^129^Xe polarization to achieve a net 1‐liter volume, which subjects inhaled from functional residual capacity. Patients remained supine on the table during the entirety of the approximately 20‐minute imaging session. Heart rate and oxygen saturation were continuously monitored with an MR‐compatible monitoring system (Nonin 7500 Pulse Oximeter, Nonin Medical Inc., Plymouth, MN).

The calibration scan was performed over a 16 s breath‐hold, during which 600 ^129^Xe free induction decays were acquired at 20 msec intervals (echo time 0.45 msec, flip angle target 20°, dwell time 37 μs, 512 points).^15^ Dedicated ventilation images were acquired during an 8.5 s breath‐hold using a multi‐slice gradient echo (GRE) sequence at 4 × 4 × 15 mm^3^ resolution (TR/TE = 7.65/3 msec, 10° flip angle, BW = 170 Hz/pixel).^16^ Subsequent 3D images of ventilation, barrier uptake, and RBC transfer (the latter two of which constitute the “dissolved phase”) were then acquired during a 15 s breath‐hold using an interleaved radial acquisition with an effective repetition time (TR) of 15 msec, gas/dissolved‐phase flip angle of 0.5°/20° and 1000 radial views per phase (2000 total views). The signal was acquired at an echo time (approximately 0.47 msec) that allowed the two dissolved‐phase compartments to be decomposed using the 1‐point Dixon method.^17^ This process generated 3D images of the gas, barrier, and RBC components with a nominal isotropic resolution of 6.25 mm.

Although both ^129^Xe acquisitions provide ventilation images, the 3D dissolved‐phase sequence is significantly undersampled relative to the 2D GRE acquisition and may not resolve smaller defects.^16^ For this reason, the quantitative ventilation metrics were derived from the 2D GRE acquisition. Dedicated ventilation images as well as gas‐exchange images were deemed acceptable for analysis if the gas‐phase SNR was ≥5 based on previous work by He et al.^18^

### Anatomical ^1^H MRI Scan Parameters

To facilitate quantitative analysis of the gas exchange and dedicated ventilation ^129^Xe images, two separate anatomical ^1^H MRI scans were acquired. Each scan was acquired during a single breath‐hold of a 1‐liter airbag dose in order to match lung inflation volume with their corresponding ^129^Xe scans. An isotropic radial image was acquired with an FOV matched to the gas exchange ^129^Xe scan, and a 2D steady‐state fast spin‐echo scan was acquired with an FOV and number of slices matched to the dedicated ventilation ^129^Xe scan.

### Image Analysis

Dedicated ^129^Xe ventilation images were rendered into quantitative maps by rescaling by their top percentile of intensities and assigning each voxel into one of six classification “bins” using pre‐existing thresholds derived from a healthy reference cohort described previously.^19^ The percent of voxels falling in the lowest bin was designated the “ventilation defect percent,” or VDP, and the percent of voxels in the next‐lowest bin was designated the “low ventilation percent,” or LVP. Ventilation deficit was quantified as the sum of VDP and LVP, or “ven_def + low_.” This metric was chosen to capture potentially meaningful changes in partially obstructed (i.e., hypoventilated) as well as fully defected regions, an approach suggested previously by Myc et al.^20^ However, to provide a bridge to the existing literature, we also analyzed the change in the well‐established VDP metric.

Dissolved‐phase images were divided on a voxel‐by‐voxel basis by the gas‐phase intensities from the same acquisition in order to create normalized images of barrier uptake and RBC transfer. These were rendered into quantitative maps within the ventilated region of the thoracic cavity (i.e., the region within the thoracic cavity mask not classified as VDP on the corresponding gas‐phase image), thereby omitting voxels where the ventilation signal is most likely to be noise‐driven. Normalization to gas‐phase was performed on a voxel‐by‐voxel basis and then averaged to get the mean signal value, which was then divided by the corresponding mean value of the healthy reference population to produce the metrics bar_%ref_ and RBC_%ref_.^21^

### Pulmonary Function Tests

Spirometry and plethysmography were referenced using the Crapo/Hsu prediction equations.^22^, ^23^ Total lung capacity (TLC) and residual volume (RV) were measured using plethysmography for all but one subject. Diffusing capacity of the lung for carbon monoxide (DL_CO_) was measured using Vmax® Encore System (CareFusion, Yorba Linda, CA) and referenced using the Gaensler‐Smith equation.^24^ All tests met the technical criteria set by the American Thoracic Society and European Respiratory Society.^25^, ^26^, ^27^

### Secondary Outcome Measures

Secondary outcome measures included the COPD Assessment Test (CAT),^28^ St. George's Respiratory Questionnaire (SGRQ),^29^ and the modified Borg Dyspnea Scale and 6‐minute walk test (6MWT).^30^ These measurements were performed at both the pre‐ and post‐treatment ^129^Xe MRI visits.

### Statistical Analysis

A paired Student's *t*‐test was used to assess changes in ven_def + low_ and conventional clinical measures following therapy. Pearson's correlation coefficient was used to assess the association between changes in ^129^Xe ventilation and PFTs/secondary outcomes, and to evaluate baseline measures of interstitial barrier and PFTs as potential predictors of ^129^Xe ventilation response to therapy. All statistical analysis was performed using R version 3.6.0.^31^ A *P*‐value of <0.05 was considered statistically significant. Note that this definition of significance is agnostic to a specific threshold for clinically meaningful changes in ventilation on ^129^Xe MRI, as such a threshold has yet to be empirically established.^32^

## Results

**FIGURE 1:**
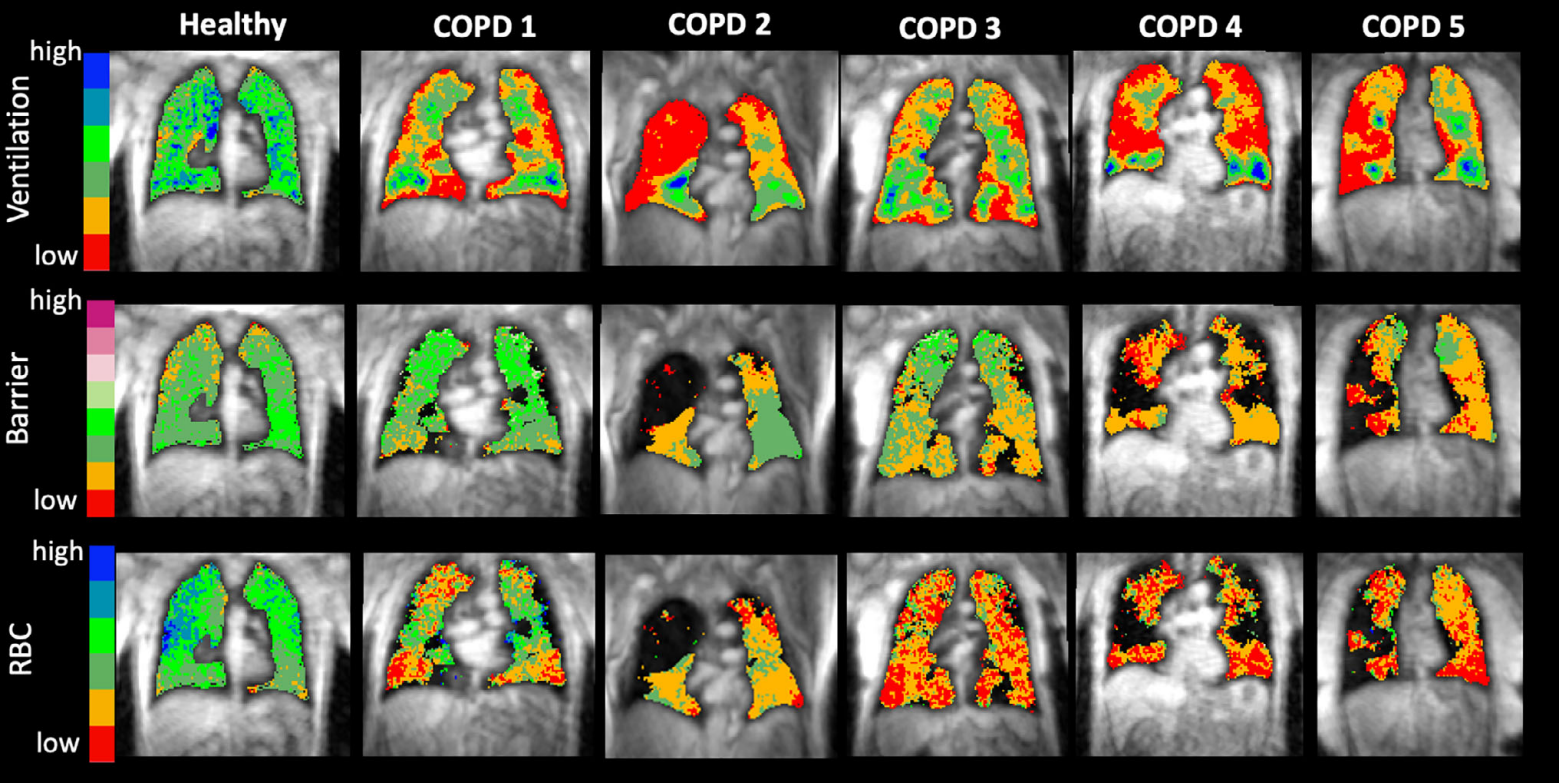
Example baseline^129^Xe magnetic resonance imaging (MRI) maps of ventilation, barrier uptake, and red blood cell (RBC) transfer from five chronic obstructive pulmonary disease (COPD) subjects and a healthy subject for reference. All maps are overlaid onto an anatomical proton MRI scan for reference. On the ventilation map, the lowest two of the color bins correspond to the ven_def + low_ measurement. Barrier and RBC images are also binned to aid in visual interpretation. Note that black areas are associated with regions of ventilation defect where analysis of barrier and RBC is not possible. The healthy subject underwent informed consent and imaging as part of another study in our lab and is presented here solely for visual context.

Twenty subjects were initially recruited. One subject was excluded from data analysis due to poor image signal‐to‐noise ratio (SNR), and two elected not to return for a post‐treatment visit; thus, a total of 17 subjects (8M 9F) were included in the data analysis. Clinical data and pre‐treatment pulmonary function for this study population are shown in Table 1. Nine subjects were classified as GOLD II and eight as GOLD III. Figure 1 shows images of ventilation, barrier uptake, and RBC transfer from five example COPD subjects along with a representative healthy subject for reference. These COPD subjects exhibited clear regions of ven_def + low_ (red and yellow), which were especially extensive in subjects 2, 4, and 5. Subjects 2–5 also exhibited clear regions of reduced barrier uptake (red and yellow) and all five subjects exhibited regions of reduced RBC transfer, with subjects 3–5 appearing devoid of any normal RBC transfer. Note that in the barrier and RBC maps, the dark areas within the thoracic cavity represent unventilated regions (defects) where analysis of gas exchange is not possible. Overall, subjects at baseline (i.e., prior to treatment) had ven_def + low_ of 57.8 ± 8.4%, bar_%ref_ of 73.2 ± 19.6%, and RBC_%ref_ of 36.5 ± 13.6%.

**TABLE 1:** Clinical Characteristics and Pre‐Treatment Pulmonary Function of the Study Population

Patient	Sex	Age (years)	FEV_1_% pred	FVC% pred	TLC% pred	RV% pred	DL_CO_% pred
01	F	55	29	74	124	206	42
02	F	67	33	74	113	155	52
03	F	71	75	98	84	64	51
04	M	64	66	97	88	72	39
05	F	58	29	66	104	150	36
06	M	66	61	77	92	121	75
07	F	64	61	91	96	98	76
08	M	74	64	96	101	104	34
09	M	69	43	76	96	134	71
10	F	61	51	73	95	123	64
11	M	59	68	94	95	98	66
12	F	66	48	60	96	143	104
13	F	66	42	89	125	173	32
14	F	67	57	90	129	167	67
15	M	67	38	67	NA	NA	54
16	M	58	45	97	112	144	56
17	M	61	70	90	99	121	64
Mean		64.3	52	83	103	127	58
SD		4.9	15	13	12	29	19

All pulmonary function tests expressed as percent predicted (%).

DL_CO_ = diffusion capacity of the lung for carbon monoxide; FEV_1_ = forced expiratory volume in 1 second; FVC = forced vital capacity; RV = residual volume; TLC = total lung capacity.

### Change in Measurements Following Therapy

**FIGURE 2:**
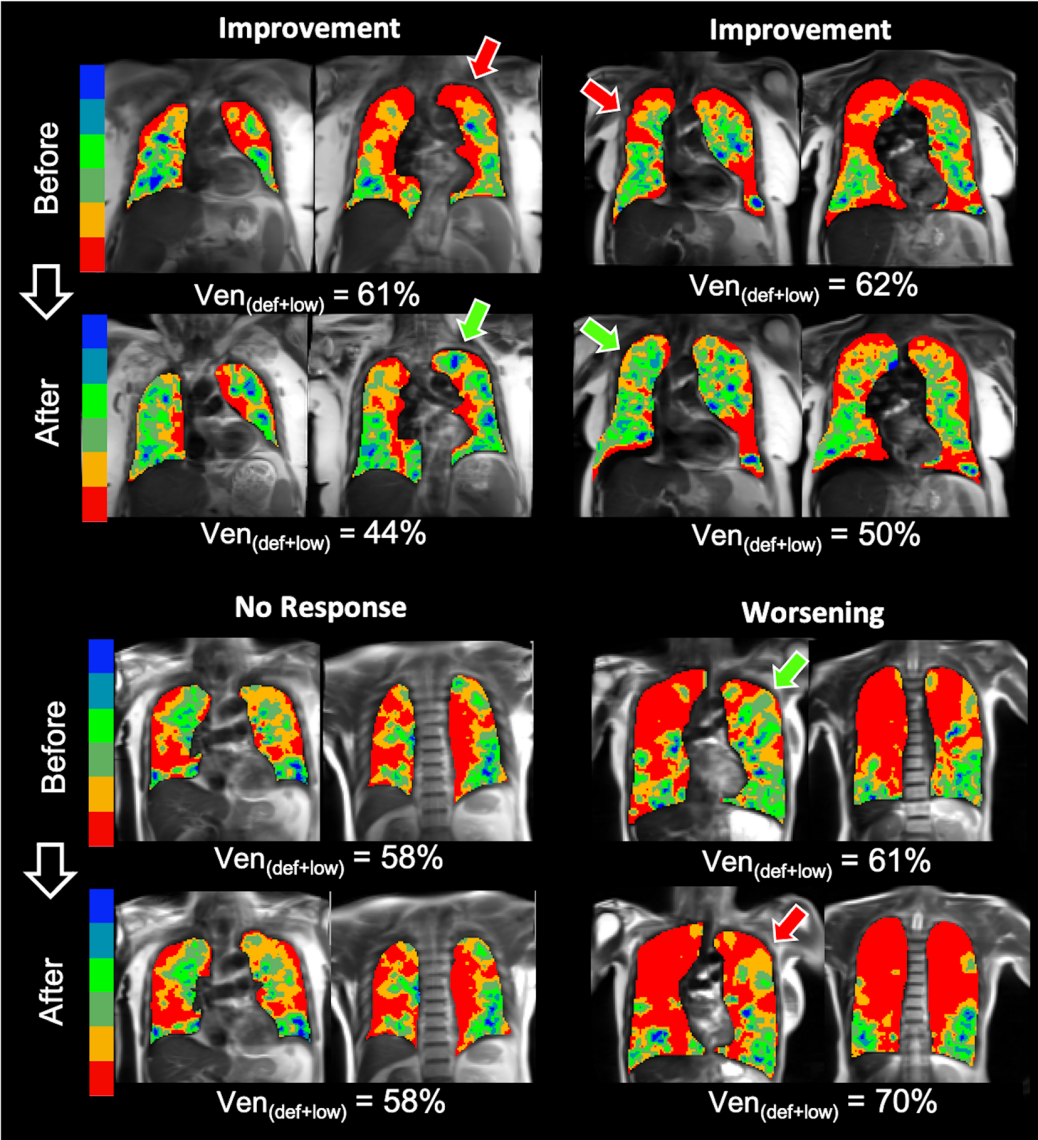
^129^Xe magnetic resonance imaging (MRI) ventilation maps before and after therapy from four representative subjects. The subjects in the top row showed improved (i.e., reduced) ven_def + low_ following therapy, while subjects in the bottom row exhibited no change (left) or worsening (right). Arrows indicate prominent regions where ventilation changed following therapy.

Representative ^129^Xe ventilation images from subjects with a range of therapeutic responses are shown in Fig. 2. Some subjects exhibited a clear decrease in ven_def + low_ (top row, left and right) after LABA/LAMA; green arrows indicate newly ventilated regions. However, other subjects showed no change in ven_def + low_ or ventilation pattern (bottom row, left), while others showed an increase in ven_def + low_ with newly emergent ventilation defects (bottom row, right), indicated by the red arrow.

**FIGURE 3:**
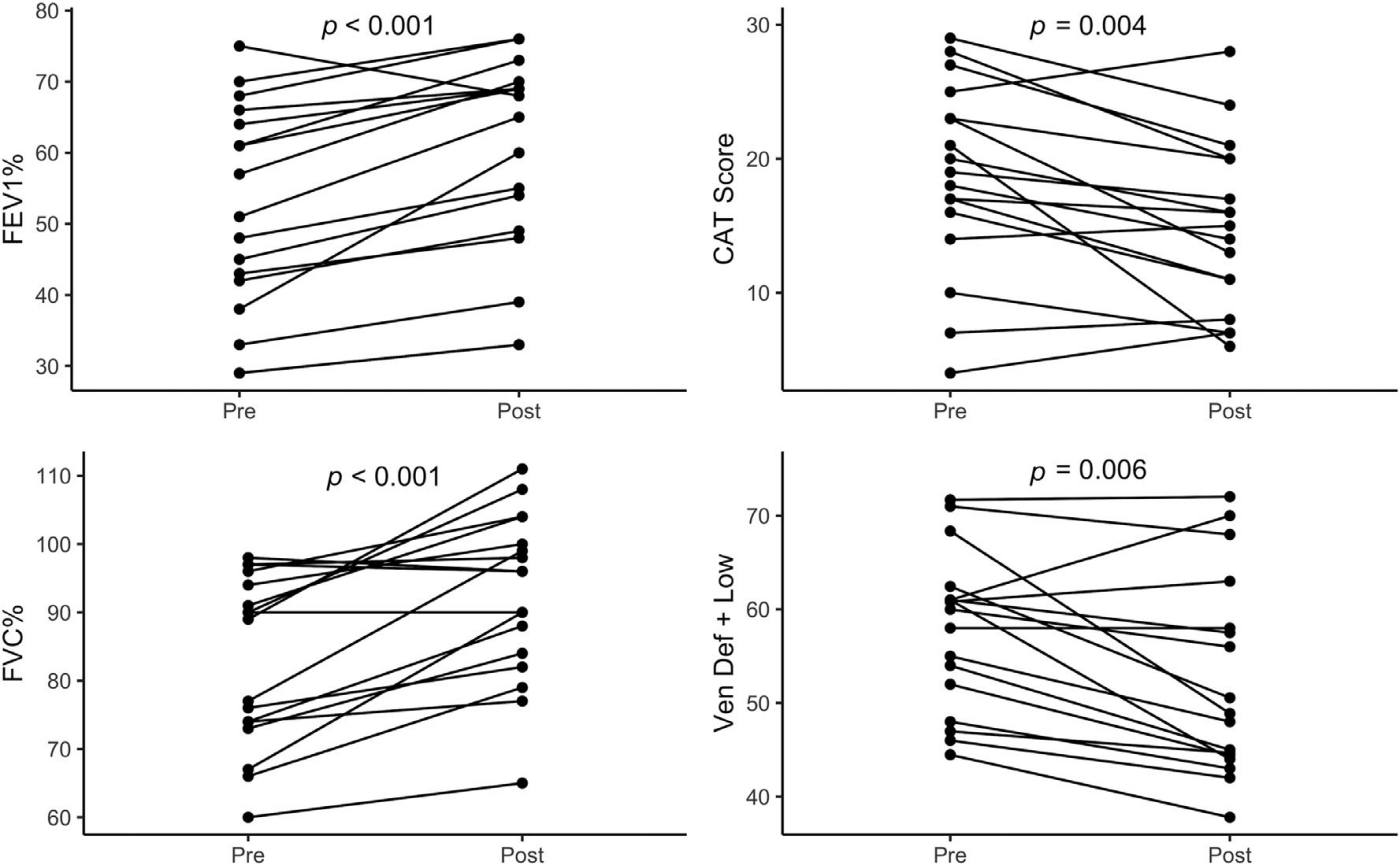
Clinical and ventilation metrics with significant changes following therapy. Each quadrant contains one of the four metrics that changed significantly following therapy. Values from each individual subject are shown before and after therapy, connected by a line. Significance: FEV_1_%, *P* < 0.001; CAT, *P* = 0.004; FVC%, *P* < 0.001, ven_def + low_, *P* < 0.006).

In the overall study population, ventilation improved, with ven_def + low_ decreasing from 57.8 ± 8.4% to 52.5 ± 10.6% following treatment (*P* < 0.05), as did VDP from 33.7 ± 8.9% to 29.5 ± 11.4% (*P* < 0.05). Barrier uptake did not change, with bar_%ref_ values of 73.2 ± 19.6% before and 75.1 ± 20.6% after treatment (*P* = 0.23). Similarly, RBC_%ref_ did not change, with values of 36.5 ± 13.6% before and 35.1 ± 14.0% after treatment (*P* = 0.21). Therapy also improved several conventional pulmonary function metrics: specifically, FEV_1_% and FVC% increased significantly following therapy (*P* < 0.05 in both cases). However, most metrics did not change significantly, including FEV_1_/FVC (*P* = 0.10), DL_CO_% (*P* = 0.80), TLC% (*P* = 0.16), or RV% (*P* = 0.24). Among clinical measures, CAT score decreased significantly (*P* < 0.05), but there was no significant change in Borg dyspnea score (*P* = 0.28), 6MWT (*P* = 0.12), or SGRQ (*P* = 0.44). The four metrics that changed significantly after therapy, including ven_def + low_, FEV_1_, FVC, and CAT score, are shown in Fig. 3.

The absolute change in ven_def + low_ after therapy was not significantly correlated with changes in FEV_1_% (*P* = 0.15), FVC% (*P* = 0.81), FEV_1_/FVC (*P* = 0.08), TLC% (*P* = 0.50), RV% (*P* = 0.26), or DL_CO_% (*P* = 0.66), nor with changes in 6MWT (*P* = 0.60) or with any of the questionnaires, including CAT (*P* = 0.73), SGRQ (*P* = 0.45), or Borg dyspnea score (*P* = 0.91).

### Changes in ^129^Xe Ventilation, Barrier Uptake, and RBC Transfer

**FIGURE 4:**
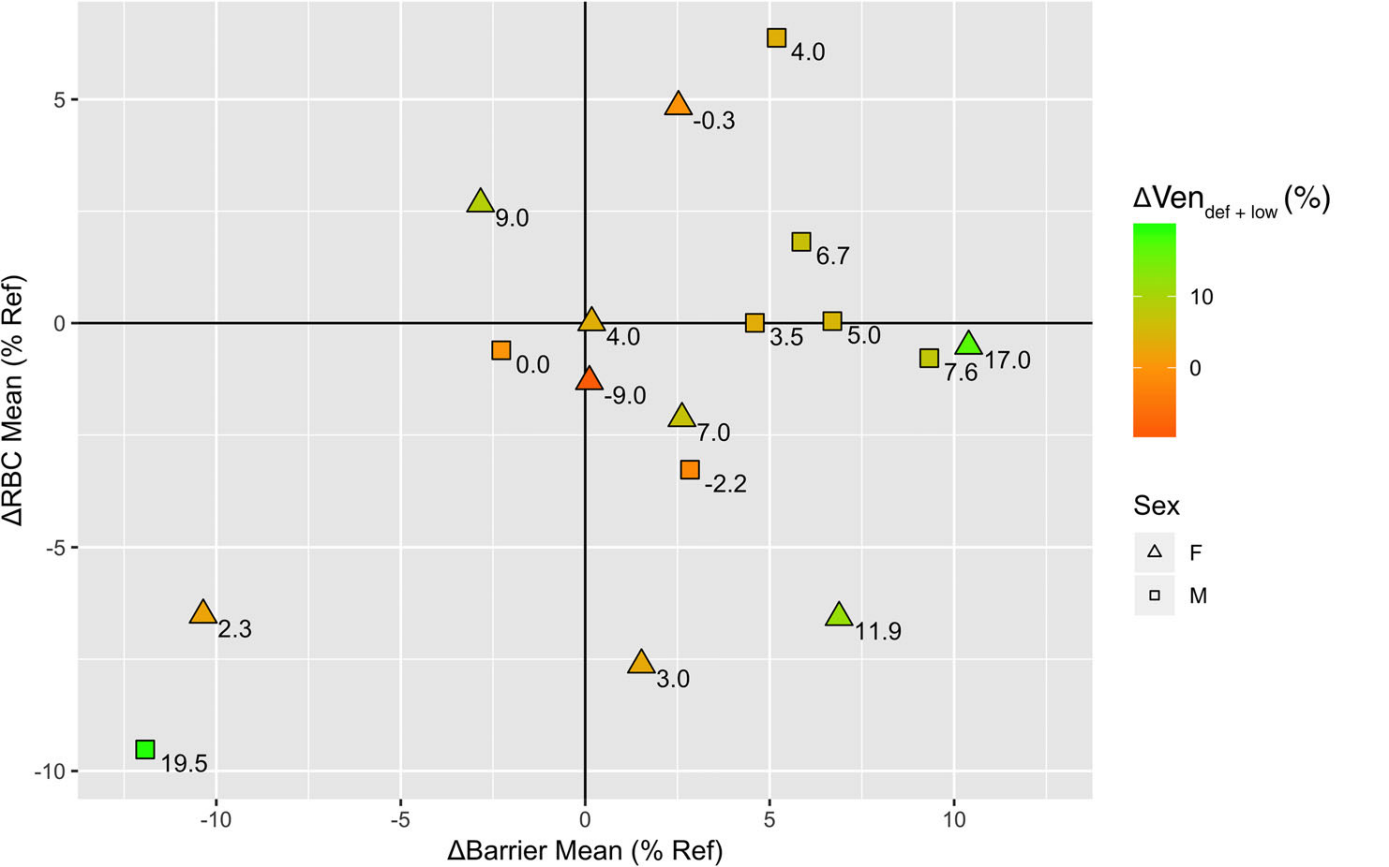
Changes in ^129^Xe gas exchange following therapy. Change in bar_%ref_ (*x*‐axis) vs. change in RBC_%ref_ (*y*‐axis) for each subject in the cohort following therapy. The value next to each point indicates the change in ven_def + low_ following therapy, where a positive value (green color) indicates a decrease in the amount of ventilation defect.

**FIGURE 5:**
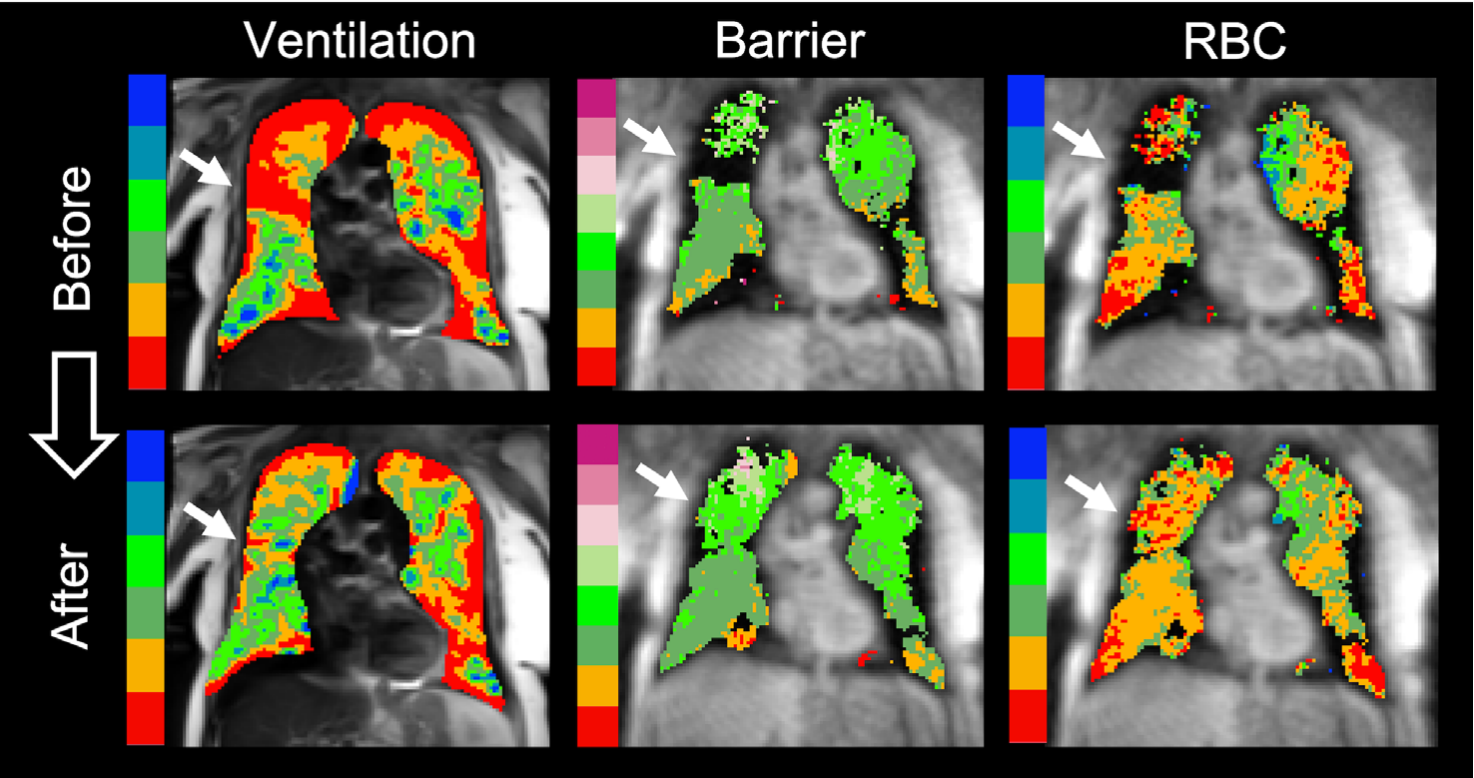
A newly ventilated region with poor red blood cell (RBC) transfer. Example of a subject where a region of improved ventilation following therapy (white arrows) revealed areas of normal barrier uptake but poor RBC transfer.

Absolute changes in ^129^Xe ven_def + low_, bar_%ref_, and RBC_%ref_ following therapy are shown together in Fig. 4. Change in RBC_%ref_ is shown on the vertical axis and change in bar_%ref_ on the horizontal. Numbers next to each point (and the associated color scale) indicate the improvement (or worsening) in ven_def + low_ for that particular subject. Only 3 of 17 (17.6%) subjects exhibited worsening ventilation after therapy as indicated by increasing ven_def + low_. Therapy caused mean barrier uptake to increase in 13/17 (76.5%) of subjects (i.e., right of the origin), whereas it revealed a decrease in RBC_%ref_ (below the origin) in 10/17 (58.8%) subjects. The trend toward increased mean barrier uptake with a concomitant decrease in mean RBC transfer is illustrated by the plurality of subjects (7/17, 41.2%) falling in the lower‐right quadrant of Fig. 4. Indeed, only one subject (1/17, 5.9%) is in the upper‐left quadrant, indicating decreased barrier signal but increased RBC signal. An example of a subject with increased barrier uptake and decreased RBC transfer following therapy is shown in Fig. 5.

### Baseline Measurements Associated with ^129^Xe Ventilation Change

**FIGURE 6:**
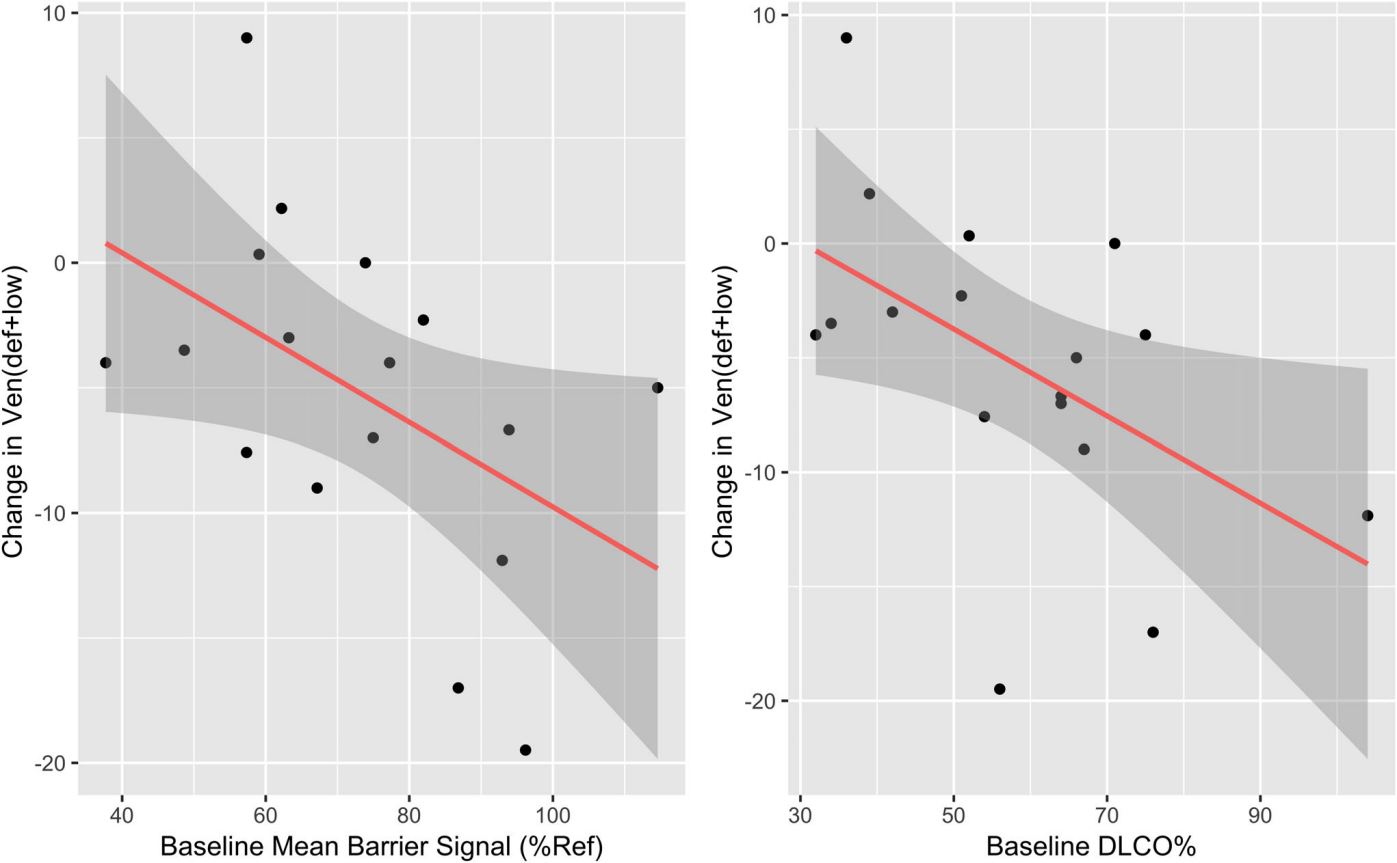
Barrier uptake and diffusing capacity of the lung for carbon monoxide (DL_CO_) at baseline are correlated with ventilation change. Change in ven_def + low_ was correlated with both baseline bar_%ref_ (shown at left; *r* = 0.49, *P* = 0.048) and DL_CO_% (right; *r* = −0.52, *P* = 0.032). None of the other baseline pulmonary function tests (PFTs) or clinical markers under consideration were significantly predictive of change in ven_def + low._ Linear regression line is shown with the shaded area representing the 95% confidence interval of the model.

As seen in Fig. 6, two metrics measured at baseline were found to be associated with post‐therapy changes in ven_def + low_: bar_%ref_ (*r* = −0.49, *P* < 0.05) and DL_CO_% (*r* = −0.52, *P* < 0.05), although RBC_%ref_ was just short of the threshold of significance (*r* = −0.47 [95% CI −0.77 to 0.01], *P* = 0.057). None of the other baseline PFTs or clinical markers under consideration were significantly associated with change in ven_def + low_, including FEV_1_% (*P* = 0.44), FVC% (*P* = 0.40), FEV_1_/FVC (*P* = 0.43), TLC% (*P* = 0.69), RV% (*P* = 0.82), CAT (*P* = 0.92), SGRQ (*P* = 0.80), 6MWT (0.83), or Borg dyspnea score (*P* = 0.08).

## Discussion

In this study, we found that each patient in this cohort of GOLD II/III COPD patients exhibited significant ventilation and RBC transfer defects, with barrier uptake that ranged from low to normal. As expected given our exclusion criteria, no subjects exhibited the high barrier uptake that has been previously associated with interstitial lung disease.^8^, ^9^, ^10^ After 2 weeks of LABA/LAMA therapy, regional ventilation was significantly improved across the cohort, reflected in decreasing ven_def + low_. These changes were also accompanied by significant improvements in FEV_1_%, FVC%, and CAT score. The improvement in ventilation enabled barrier uptake and RBC transfer to be analyzed in previously inaccessible regions of the lung. This revealed modestly higher barrier uptake after LABA/LAMA, but also exposed regions of reduced or absent RBC transfer. Ventilation improved in most but not all patients, with the degree of response being associated with baseline ^129^Xe barrier uptake and DL_CO_. Specifically, patients who had a more preserved level of barrier uptake, or a higher DL_CO_, exhibited greater improvement in ^129^Xe ventilation following therapy. This is consistent with the finding of Han et al that COPD subjects with higher DL_CO_ were more likely to exhibit bronchoreversibility.^33^

Reduced ^129^Xe barrier signal and DL_CO_ are both consistent with an emphysema‐predominant COPD phenotype in which the alveolar septa have been destroyed.^34^ This reduces the alveolar surface area available for gas diffusion into the blood and leads to airway collapse.^34^ Conversely, patients with relatively preserved measures of barrier uptake and DL_CO_ may have airway obstruction that is caused by a bronchitis‐predominant phenotype.^34^ It is this subset who appeared more likely to respond to the LABA/LAMA treatment as measured by ^129^Xe ventilation MRI. This is further supported by the observation that mean barrier uptake increased after treatment, suggesting that newly exposed regions of the lung had preserved surface area for gas exchange. This is consistent with earlier findings by Baldi et al, in which bronchodilation in COPD was associated with a small but significant increase in DL_CO._ ^35^

By contrast, the consistently poor RBC transfer in this cohort both before and after therapy is striking mean ^129^Xe RBC transfer signal actually tended to decrease following therapy. Since ^129^Xe RBC transfer is a measure of raw RBC signal normalized by local ventilation, the observed decrease may be the result of newly ventilated regions exposing an underlying architecture with significant microvascular abnormalities.^36^, ^37^, ^38^ Our observations of increased barrier uptake coupled with decreasing RBC transfer after dual bronchodilator therapy suggest that these underlying regions had relatively preserved surface area for gas exchange, but significant underlying vascular abnormalities or lack of capillary blood volume. The finding that therapy‐induced redistribution of ventilation revealed additional regions of impaired gas exchange indicates a need for further studies to determine whether additional treatments targeting the pulmonary vasculature can recover more functionality in those regions.

These results suggest that a comprehensive assessment of therapeutic response in COPD requires functional measurements of gas exchange beyond those afforded by measurements of airway limitation and obstruction alone. Thus, ^129^Xe MRI is uniquely positioned to address this question by not only visualizing regional ventilation response but also revealing the functionality of the underlying tissue and vasculature that has been exposed.

In addition to improved regional ^129^Xe ventilation, we observed significant improvements in FEV_1_%, FVC%, and CAT scores. This is consistent with prior randomized controlled trials of the LABA/LAMA inhaler used in this study in moderate‐to‐severe COPD patients. Specifically, the PINNACLE‐1 and PINNACLE‐2 trials of glycopyrrolate/formoterol showed that after 24 weeks of therapy, the pre‐dose trough FEV_1_ increased by 153 mL and 105 mL respectively.^39^ In another study, peak FEV_1_ increased by approximately 300 mL after 7 days of therapy.^40^ Similarly, after 24 weeks of glycopyrrolate/formoterol MDI, prior studies have shown a change in CAT score of −3.^41^ This is similar to the change of −3.7 that we observed.

Thus, while numerous conventional metrics are capable of measuring a significant response to LABA/LAMA therapy, ^129^Xe MRI is able to measure that response regionally, and to directly observe the underlying gas exchange characteristics of newly ventilated regions. Of note is the relatively poor RBC transfer in newly ventilated regions. This appears to suggest that truly improving the trajectories of these patients will require addressing the underlying vascular abnormalities that currently prevent these newly ventilated regions from contributing meaningfully to gas exchange.

## Limitations

Our study has several limitations. First, our sample size is relatively small and the study was conducted over a short time period. A larger population would allow us to better detect overall patterns of changes in gas exchange function following therapy, and enable multivariate models directly comparing DL_CO_ and ^129^Xe as predictors of outcomes. A longer follow‐up period would enable the characterization of transient vs. long‐term changes in gas exchange following treatment, possibly as a result of ventilation‐perfusion matching. Second, CT was not acquired as part of the protocol. A contemporaneous CT could provide confirmation of disease phenotype and aid in the interpretation of ^129^Xe findings, as illustrated by recent work by Myc et al.^20^ Third, the repeatability of ^129^Xe MRI ventilation measurements in COPD has not been well characterized, limiting our ability to determine a clinically meaningful change in ventilation metrics. Previous studies have showed high repeatability for ^129^Xe measurements of VDP in asthma^4^ and cystic fibrosis,^42^ and analogous studies are needed for COPD. Further, while the RBC to barrier ratio is known to be more robust to lung inflation than those measurements taken individually,^43^ it is not possible to tell whether changes in this ratio are the result of changes in RBC, barrier, or both; thus we have elected here to report the two as separate measurements. Finally, in order to compare ^129^Xe MRI with conventional clinical metrics, we employed measures derived from the whole lung. However, additional insights may be gained by more regional analysis such as illustrated by Matin et al, who evaluated lobar correlations between CT measures of emphysema with ^129^Xe ventilation and apparent diffusion coefficient (ADC). A similar approach could be used with dissolved‐phase measures of gas exchange.^44^

## Conclusion

In this study, LABA/LAMA therapy tended to preferentially improve ventilation in those subjects with relatively preserved measures of ^129^Xe barrier uptake and DL_CO_. However, even in subjects with improved ventilation, newly ventilated lung regions often revealed persistent ^129^Xe RBC transfer defects, an aspect of LABA/LAMA therapy response that is opaque to spirometry. Taken together, these results add to the body of knowledge regarding COPD phenotypes and indicate a possible role for ^129^Xe gas transfer MRI as a tool for both patient selection and measuring treatment response in future COPD clinical trials. As we develop therapies that demonstrably improve not only ventilation but also RBC transfer, ^129^Xe MRI may ultimately develop into a tool that can guide individualized patient care.

## Conflict of Interest

D.G.M. is a consultant with Polarean Imaging, plc. B.D. is a founder and shareholder with Polarean Imaging, plc.

## Author Contributions

B.D. and Y.‐C.H. designed and implemented the study. D.G.M., E.M.C., Z.W., and E.A.B. collected the data. D.G.M., E.M.C., Z.W., E.A.B., and J.L. were responsible for data analysis. All authors contributed to the writing of the manuscript.
